# Quality of end-of-life care in patients with dementia compared to patients with cancer: A population-based register study

**DOI:** 10.1371/journal.pone.0201051

**Published:** 2018-07-30

**Authors:** Lisa Martinsson, Staffan Lundström, Johan Sundelöf

**Affiliations:** 1 Department of Radiation Sciences, Umeå University, Umeå, Sweden; 2 Department of Palliative Medicine, Stockholms Sjukhem Foundation, Stockholm, Sweden; 3 Department of Oncology-Pathology, Karolinska Institutet, Stockholm, Sweden; 4 Betaniastiftelsen (non-profit organisation), Stockholm, Sweden; Taipei Veterans General Hospital, TAIWAN

## Abstract

**Introduction:**

Globally, dementia is one of the leading causes of death. Given the growing elderly population in the world, the yearly number of deaths by dementia is expected to increase. Patients dying from dementia are reported to suffer from a burden of symptoms similar to that of patients with cancer, but receive less medication against symptoms, have a lower probability of palliative care planning and seldom have access to specialised palliative care. Studies investigating the quality of palliative care in dementia are scarce. The aim of this Swedish national study was to compare the quality of end-of-life care between patients with dementia and patients with cancer regardless of place of care.

**Methods:**

Thirteen end-of-life care quality indicators collected by the Swedish Register of Palliative Care (SRPC) were compared between patients dying from dementia and patients dying from cancer. Data were collected from deaths occurring in nursing homes, hospitals, specialised and general palliative home care, and palliative in-patient units during a three-year period (during March 2012 to February 2015). Analyses were performed using a multivariable logistic regression model, adjusted for age and gender. A subgroup of patients with Alzheimer’s disease was identified and compared to patients with other and unspecified types of dementia.

**Results:**

A total of 4624 deaths from Alzheimer’s disease, 11 804deaths from other dementia diagnoses and 51 609 deaths from cancer were included. For six of the 13 quality indicators examined (prescription of PRN drugs against nausea and anxiety, information and bereavement support offered to next of kin, pain assessment and specialised palliative care consultations), poorer outcomes were shown for the dementia group in comparison to the cancer group. Two outcomes (prevalence of pressure ulcers and fluid therapy during the last 24 hours in life) showed better outcomes for the dementia group. The outcomes for the 13 quality indicators were similar for patients with Alzheimer’s disease compared to patients with other and unspecified types of dementia.

**Conclusions:**

The findings in this study indicates that patients dying from Alzheimer’s disease and other types of dementia receive a poorer quality of end-of-life care concerning several important end-of-life care areas when compared to patients dying from cancer. Guidelines for end-of-life care in Sweden cannot explain or justify these differences. Further studies are needed to find possible ways to improve end-of-life care in the large and growing group of patients dying from dementia.

## Introduction

According to the World Health Organization, dementia was the seventh cause of death globally in 2015, causing 1.54 million deaths, twice as many as in 2000 [1]. The number of deaths due to dementia is expected to increase globally in the future due to a growing population [2]. In Sweden, according to the National Board of Health and Welfare, around 8 000 people die from dementia yearly, which corresponds to approximately 9% of all deaths [3].

Patients with advanced dementia are reported to have a high burden of symptoms [4], similar to that of patients with advanced cancer [5], but often do not have access to specialised palliative care [6–8]. In Sweden, over 90% of patients enrolled in specialised palliative care have a cancer diagnosis [9]. One proposed explanation for this difference is that dementia is not fully recognised as a terminal illness, and because of that, the need for palliative care in end-of-life is not identified [10–12]. Another important explanation is that palliative care for patients with dementia should be continuous, according to European guidelines [13], suggesting that consulting of specialised palliative care teams without physical transferring of the patients should be chosen prior to referral to a specialised palliative care unit when possible. Recognising end of life has been proposed as a key requirement for good end-of-life care for patients with dementia [14].

Five studies comparing palliative care outcomes between patients with dementia and patients with cancer were found in the literature: McCarthy et al. compared 170 patients with dementia to 1513 patients with cancer in a population-based regional study [5], Ahronheim et al. compared 80 patients with dementia to 84 patients with cancer in a hospital setting [15] and Mitchell et al. compared 1609 patients with dementia to 883 with cancer in a nursing home setting [10]. Chen et al compared outcomes during the last year of life for 908 patients with dementia and 1816 patients with cancer and found that hospitalisation and life-sustaining interventions including cardiopulmonary resuscitation and tube feeding were more common in the dementia group [16]. Huang et al examined outcomes for 7111 patients with cancer and dementia compared to 28 444 patients with cancer and without dementia. The patients with cancer and dementia were more likely to be hospitalized and to undergo invasive procedures in the last months of life, but less likely to receive hospice care [17].

Two other studies comparing palliative care outcomes between patients with and without dementia were found: Evers et al. compared 279 patients with dementia with 24 control patients in chronic care facilities [8] and Sampson et al. compared 35 patients with dementia with 65 patients without dementia in a hospital setting [7]. In these studies, patients with dementia were found to be less likely to receive analgesics [15] and prescriptions of medications for symptom management [7]. They had a lower probability of palliative care planning, such as do-not-resuscitate and do-not-hospitalise orders [10], and a higher probability of tube feeding [10,15] and antipsychotic medication [10]. Patients with dementia had greater needs for home assistance and social services when compared to patients with cancer, but were reported to see their general practitioner less often [5]. No studies comparing the quality of end-of-life care between patients with Alzheimer’s disease and other dementia diagnoses have been found in the literature.

One study compared patients with dementia that did or did not access palliative care: Chen et al found that patients with dementia who accessed palliative care were less likely to receive invasive treatment with for example haemodialysis or cardiopulmonary resuscitation compared to patients with dementia that did not access palliative care, but more often received tube feeding. For a subgroup of patients that had both dementia and cancer, access to palliative care was associated with fewer life sustaining treatments [18].

The Swedish Register of Palliative Care (SRPC) gathers national data about quality of end-of-life care for dying patients in Sweden, regardless of diagnosis, age or care setting. Data collection includes information provided to patient and family, decision-making capacity, symptom relief, prescribed drugs, preferred place of death and support to the family [19]. Around 60 000 deaths per year have been reported to the SRPC during the last five years, corresponding to approximately 65% of all deaths in Sweden during that period [20]. Data from the SRPC have previously been used to compare the quality of end-of-life care between heart diseases and cancer [21], stroke and cancer [22] and oxygen-dependent interstitial lung disease and lung cancer [23]. Patients dying from cancer received better end-of-life care compared to the other diagnosis groups.

### Aim

The aim of this study was to compare the quality of end-of-life care between patients with dementia and patients with cancer on a population-based level regardless of place of care.

## Methods

### Planning and setting

The study and control groups were identified via the SRPC. During 2015, data from 66% of all deaths in Sweden were gathered by the SRPC [24]. The SRPC collects data with a web-based end-of-life questionnaire (ELQ), which is completed by healthcare staff after the death of a patient. Items focus on the last week of life and concern several aspects of the quality of end-of-life care. The ELQ was developed based on the principles of a good death proposed by the British Geriatrics Society [25]. Stored data are matched weekly with the central population register for validation purposes [19]. The validity of the ELQ has been examined in a specialised palliative care setting and was shown to vary between different items, but with overall improvements in an updated version of the ELQ [26,27].

The SRPC database also contains individual-level information about the cause of death according to the 10th revision of the International Classification of Diseases (ICD-10). These data are retrieved from the Cause of Death Register at the Swedish National Board of Health and Welfare.

Inclusion criteria for the study group were as follows: data were reported to the SRPC, patient deceased during March 2012 to February 2015, 18 years or older, no forensic port-mortem examination was planned, death was reported as expected and dementia was the underlying cause of death according to the Cause of Death Register. Exclusion criterion was report of cancer as contributing cause of death in the Cause of Death Register data (ICD-10 C00–D48, including subcategories). Dementia was classified according to the ICD-10 (F00, F01, F02, F03 and G30, including subgroups). Subgroups of patients with Alzheimer’s disease (ICD-10 F00 and G30, including subgroups) and other and unspecified causes of dementia (ICD-10 F01, F02 and F03, including subgroups) were identified.

The control group had the same inclusion criteria as the study group but with cancer as underlying cause of death according to the Cause of Death Register. Exclusion criterion for the control group was report of dementia as contributing cause of death in the Cause of Death Register data. Cancer patients were chosen as controls because several studies that have compared end-of-life care quality between patients with cancer and other diseases using SRPC data have shown that patients with cancer generally receive better end-of-life care compared to patients with other diseases [21–23], thus enabling a comparison with “best standard” end-of-life care. The control group of cancer patients was also chosen because Swedish cancer deaths have better coverage in the SRPC compared to cardiovascular diseases and lung diseases [24].

The following quality indicators based on items collected by the ELQ in the SRPC database were analysed: documented decision by the responsible physician to shift treatment/care to end-of-life care; pressure ulcers at death; assessment of oral health during the last week of life; someone present at the moment of death; information to next of kin about transition to end-of-life care; offer of a follow-up talk to next of kin after death of the patient; fluids via enteral tube or intravenously during the last 24 hours of life; assessment of pain and other symptoms during the last week of life; prescriptions of PRN drugs against pain, anxiety and nausea; and consultation of a specialised palliative care team during the last week of life. A robust validity for items about symptom prevalence and symptom alleviation has not been established in the SRPC [27], and these items were therefore not deemed suitable as quality indicators in this study. We did not include the item about place of death in line with the patient’s last stated wishes because of the uncertain nature of data collection for the dementia group for this item.

### Power analysis

We estimated that we would be able to identify at least 800 patients per subgroup and year. To be able to detect a 5 percentage point difference in the outcomes between subgroups, with significance level below 0.05 and power of 0.8, the study would require 1600 patients per subgroup. The study period was set from March 2012 to February 2015, a period when no changes were made to the ELQ.

### Data analysis

Chi-square test (gender and place of death) and t-test (age) were used to compare groups and subgroups. The outcomes were dichotomised after exclusion of the “Don’t know” answers. When analysing whether a specialised palliative care team was consulted, only patients cared for outside specialised palliative care were included. Patients with no next of kin were excluded from the analyses regarding next of kin. The outcomes were then analysed with logistic regression, with cause of death (dementia vs cancer) as independent variable. A model adjusted for age and gender was developed. A limit of 5 percentage point for clinically significant differences in outcomes between subgroups was chosen.

### Ethics

The working procedure and study design were approved by the Regional Ethical Review Board in Umeå, Sweden (registration number 2017/11-31).

## Results

A total of 19 105 adult deaths from dementia and 56 113 adult deaths from cancer were identified in the SRPC database. For none of these had a forensic investigation been performed. Death was reported as expected for 17 289 of the patients with dementia and for 53 369 of the patients with cancer. In the dementia group, 861 patients had cancer reported as a contributing cause of death and were excluded. In the cancer group, 1760 patients had dementia reported as a contributing cause of death and were excluded. 16 428 dementia cases and 51 609 cancer cases remained in the study (Table 1). The dementia group was subdivided into 4624 patients who had died from Alzheimer’s disease and 11 804 who had died from other dementia diagnoses or unspecified dementia (Table 2).

**Table 1:** Gender, age, and place of death for the dementia and cancer groups.

Patient characteristics		Dementia (16 428)	Cancer (51 609)
Gender (n) women/men p < 0.001		11 169 (68.0%)/ 5259 (32.0%)	25 273 (49.0%)/ 26 336 (51.0%)
Mean age (years) p < 0.001		86.7	74.4
Median age (years)		88.0	76.0
Age range (years)		41–110	18–106
Distribution of place of death (n) p < 0.001	Permanent stay nursing home	14 564 (88.7%)	5080 (9.8%)
	Short-term nursing home	481 (2.9%)	5553 (10.8%)
	Hospital	971 (5.9%)	14 791 (28.7%)
	Hospice or palliative in-ward unit	60 (0.4%)	14 533 (28.7%)
	Own home with support from specialised palliative home care	43 (0.3%)	7783 (15.1%)
	Own home with support from general palliative home care	260 (1.6%)	3605 (7.0%)
	Other place	49 (0.3%)	264 (0.5%)

**Table 2:** Gender, age, place of death and cause(s) of death for the patients dying from Alzheimer’s disease compared to other dementia diagnoses and unspecified dementia.

Patient characteristics		Alzheimer’s disease (ICD-10 F00 and G30 and subgroups) n = 4624	Other or unspecified dementia (ICD 10 F01 and F03 and subgroups) n = 11 804
Gender (n) women/men p = 0.206		3120 (67.5%)/ 1504 (32.5%)	8049 (68.2%)/ 3755 (31.8%)
Mean age (years) p < 0.001		84.9	87.4
Median age (years)		86.0	88.0
Age range (years)		47–104	41–110
Distribution of place of death (n) p = 0.006	Permanent stay nursing home	4164 (90.1%)	10 400 (88.1%)
	Short-term nursing home	128 (2.8%)	353 (3.0%)
	Hospital	228 (4.9%)	743 (6.3%)
	Hospice or palliative in-ward unit	20 (0.4%)	40 (0.3%)
	Own home with support from specialised palliative home care	12 (0.3%)	31 (0.3%)
	Own home with support from general palliative home care	56 (1.2%)	204 (1.7%)
	Other place	16 (0.3%)	33 (0.3%)

### End-of life care outcomes

Of the 13 end-of-life care quality indicators examined, six showed worse end-of-life care outcomes for patients dying from dementia when compared to patients dying from cancer (Table 3). Two indicators showed better end-of-life care outcomes for patients dying from dementia. The outcomes for the 13 quality indicators were similar for patients with Alzheimer’s disease compared to other dementia diagnoses (Table 3). The outcome with the largest difference between the dementia group and the cancer group was that a specialised palliative care team was consulted for 1.3% of patients with dementia during the last week of life as compared to 23.8% of patients with cancer.

**Table 3:** Outcomes for 13 end-of-life care quality indicators for patients dying from Alzheimer’s disease, from all causes of dementia and from cancer, and comparison between dementia group and cancer group. Odds ratios (OR) and 95% confidence intervals (CI) are reported.

	Dementia group		Cancer group	Comparison between total dementia group and cancer group, adjusted for age and gender
	Alzheimer’s disease cases	All dementia cases		
Specialised palliative care team consulted	75/4592 (1.6%)	211/16 325 (1.3%)	6970/29 293 (23.8%)	p < 0.001 OR 0.059 (CI 0.051–0.068)
Documented decision to shift to end-of-life care	3724/4131 (90.1%)	13 147/14 670 (89.6%)	44 778/48 353 (92.6%)	p < 0.001 OR 0.732 (CI 0.680–0.788)
Next of kin had received information about transition to end-of-life care	3149/4299 (73.2%)	10 925/15 134 (72.2%)	42 297/46 846 (90.3%)	p < 0.001 OR 0.353 (CI 0.335–0.373)
Next of kin had been offered a follow-up talk after death of the patient	2900/3847 (75.4%)	9943/13 462 (73.9%)	35 561/42 302 (84.1%)	p < 0.001 OR 0.693 (CI 0.657–0.732)
Pain was assessed and documented during the last week of life	1429/4472 (32.0%)	4961/15 807 (31.4%)	19 990/48 136 (41.5%)	p < 0.001 OR 0.759 (0.727–0.793)
Symptoms other than pain were assessed during the last week of life	836/4424 (18.9%)	2850/15 581 (18.3%)	10 067/46 368 (21.7%)	p = 0.001 OR 0.911 (CI 0.864–0.961)
Oral health was assessed during the last week of life	3256/4289 (75.9%)	11 588/15 149 (76.5%)	35 957/46 276 (77.7%)	p = 0.022 OR 0.943 (0.896–0.991)
Prescription of PRN drugs against pain	4320/4608 (93.8%)	15 258/16 365 (93.2%)	49 705/51 452 (96.6%)	p < 0.001 OR 0.590 (0.539–0.646)
Prescription of PRN drugs against nausea	2852/4563 (62.5%)	9996/16 205 (61.7%)	42 210/50 985 (82.8%)	p < 0.001 OR 0.403 (CI 0.385–0.422)
Prescription of PRN drugs against anxiety	3903/4585 (85.1%)	13 844/16 295 (85.0%)	47 259/51 252 (92.2%)	p < 0.001 OR 0.609 (CI 0.572–0.648)
Pressure ulcer at death	666/4575 (14.6%)	2532/16 268 (15.6%)	11 887/50 432 (23.6%)	p < 0.001 OR 0.579 (CI 0.549–0.610)
Someone present at the moment of death	4049/4548 (89.0%)	14 348/16 146 (88.9%)	44 521/51 131 (87.1%)	p < 0.001 OR 1.512 (CI 1.421–1.610)
Fluids via enteral tube or intravenously during last 24 hours of life	136/4611 (2.9%)	572/16 376 (3.5%)	7485/51 234 (14.6%)	p < 0.001 OR 0.326 (CI 0.297–0.358)

Next of kin to patients with dementia had a lower probability of receiving information about the patient’s transition to end-of-life care and of being offered a follow-up talk after death of the patient.

The dementia group showed a lower probability of documented assessment of pain during the last week of life. There was also a lower probability of having prescriptions of PRN drugs against anxiety and nausea.

The dementia group showed better outcomes regarding prevalence of pressure ulcers and probability of not receiving fluids via enteral tube or intravenously during the last 24 hours of life.

The dementia group showed statistically significant worse outcomes regarding four of the remaining outcomes (whether there was a documented decision to shift to end-of-life care, whether symptoms other than pain and oral health was assessed during the last week of life and whether there was a prescription of a PRN drug against pain during the last day in life) and a statistically significant better outcome regarding probability of not dying alone, but the differences between the groups were very small and not deemed clinically significant.

## Discussion

In this large population-based register study, we have demonstrated that patients dying from Alzheimer’s disease and other types of dementia receive a poorer quality of end-of-life care for six out of 13 examined outcomes when compared to patients dying from cancer. This is, to the best of our knowledge, the largest nationwide study to date comparing end-of-life care for patients with dementia and cancer, made possible through the SRPC.

Because of the epidemiological nature of this study, and since the outcomes are complex and the patient cohorts are broad, it is difficult to get a full picture of what causes these differences. Clearly, there are inequalities in end-of-life care between patients dying from dementia and patients dying from cancer, although the burden of symptoms can be high in both groups, justifying equal access to palliative care. Consultation of specialised palliative care services for patients with advanced dementia is not yet established as part of routine care in Sweden. The rare use of specialised palliative care for patients with dementia seen in this study was also found by Chen et al.[18]

The proportion of patients having pressure ulcers at the end of life was similar to the findings by Mitchell et al. [10], but their study also showed a lower prevalence of pressure ulcers in patients with terminal cancer compared to patients with dementia, in contrast to the findings in our study. The number of patients with dementia receiving tube feeding or intravenous fluids during the last day of life was very low (3.5%) compared to findings in the literature from several countries. In an Italian study, 20.5% received tube feeding and 66.6% intravenous hydration during the last 48 hours in life [11], a Taiwanese study found that 67.4% of dementia patients received tube feeding during the last year in life [16] and Mitchel et al from the US found that 25% of demented residents died with a feeding tube compared with 5.2% of residents with terminal cancer [10].

In the control group of patients with cancer, 14.6% received tube feeding or intravenous fluids during the last day of life. In contrary to the findings in our study, Chen et al [16] found that patients with dementia were more likely to receive tube feeding compared to patients with cancer.

Clearly, Swedish practice of tube feeding and fluids differs from several other parts of the world according to the literature. Cultural differences and differences in routines may explain different findings. Results in our study are in accordance with guidelines from the European Association for Palliative Care (EAPC) for palliative care in dementia, which state that permanent enteral tube nutrition should as a rule be avoided in dementia [13]. The Swedish national guidelines for palliative care state that fluids should rarely be administered intravenously or via tube to patients with short expected survival [28]. These guidelines are not diagnose specific and do not explain the differences seen between the groups of dementia and cancer patients. A previous version of the Swedish national guidelines for dementia care recommended that only in rare cases nasogastric tube should be used [29]. This can be a possible explanation for the low frequency of fluid therapy for patients with dementia in our study.

For some of the quality indicators measured in this study, there are target levels determined in Swedish guidelines from the National Board of Health and Welfare: oral health assessment should be performed in 90% of all dying patients regardless of diagnoses, pain should be assessed and documented during the last week of life for all patients and 98% should be prescribed PRN drugs against pain and anxiety [30]. None of the groups in this study reached those target levels, although prescription of PRN drugs against pain was close to the target, especially for patients with cancer, while pain assessment was far from the goal for both groups.

Some differences between the groups probably reflect varying care traditions and ideas about the end of life for different patient groups and diagnoses, and perhaps different care settings could learn from each other. Unlike the general image of cancer, dementia is not always identified as a deadly disease [10].

We have shown large differences in access to specialised palliative care between the patient groups studied. Although nursing homes in general have fewer resources compared to hospitals and specialised palliative care, the duration of stay is longer, which allows the staff to know the patients and to be able to individualise the care. Patients with dementia should have continuity in care [14], and therefore, could be less suitable for transferring to specialised palliative care settings compared to patients with cancer, but specialised palliative consulting teams seem to be a highly underused resource in Sweden and are also lacking or too small in some parts of the country.

Given the differences between diagnostic groups shown both in this study and in other previous studies from the SRPC [21–23], it can be questioned whether palliative care in dementia is prioritised as highly as it should be according to Swedish policy documents. In future studies on this subject, comparison of quality of end-of-life care between different care settings could be performed. Data from the SRPC enables such analyses.

### Methodological considerations

Data are retrospective and reported by the healthcare service to the SRPC. The validity of the analysed items has been examined in a specialised palliative care setting [26,27], but not in other healthcare settings. It cannot be excluded that part of the measured differences between the groups are caused by different reporting patterns to the SRPC. Some differences between the groups could be caused by different care settings or by different local routines, factors that were not explored in this study.

The method of identifying patients with dementia using the Cause of Death Registry allowed inclusion only of patients who had died from dementia, and not of those with a dementia diagnosis who had died from other causes. The proportion of patients with Alzheimer’s disease out of all identified dementia cases is lower than expected from an epidemiological perspective. A significant proportion of the cases reported as unspecified dementia probably truly represents patients with Alzheimer’s disease.

There are aspects of high quality end-of-life care for patients with dementia that are not registered in the SRPC but which would be interesting to examine using other methods, for example, do-not-resuscitate orders, do-not-hospitalise orders, acute hospitalisation or use of antibiotics in the end of life. It would also be interesting to examine symptom prevalence during the last week of life. Efforts are being made in Sweden to strengthen the validity of such data, enabling future studies on this important topic.

## Conclusion

In conclusion, the findings in this study indicate that patients dying from Alzheimer’s disease and other types of dementia receive a poorer quality of end-of-life care in a majority of important end-of-life care areas compared to patients dying from cancer.

Since patients dying from dementia have similar symptom burden as patients dying from cancer, both groups should have access to good quality end-of-life care. Guidelines for end-of-life care in Sweden cannot explain or justify the presented differences.

Further studies are needed to examine other important end-of-life care outcomes for patients with dementia, for comparison of quality of end-of-life care between different care settings, and to find possible ways to improve end-of-life care in this large and growing patient group.
